# Water: Its History, Characteristics, Hygienic, and Therapeutic Uses

**Published:** 1861-09

**Authors:** Samuel W. Francis

**Affiliations:** New York


WATER: ITS HISTORY, CHARACTERISTICS, HYGIENIC, AND THERAPEUTIC USES.—By *Samuel W. Francis*, A. M., M. D., of New York.—2. *Mythological History.*—In the mythology of the ancient classics, we find recorded singular customs, interesting in themselves; indicative of the mind of the times, and illustrative of the beauty of the imagination, when permitted to take wings and fly amid the pleasing illusions and suggestive ideas of a poetic conception. How fascinating are the tales of by-gone days, of deified creations struggling to maintain the mastery over too ambitious, impetuous youths! How sweet and plaintive the expressive pleadings of some captive female, inspiring courage in the breasts of one and all who come to succor in the time of need! And yet, amid their fairy scenes of ambrosial life and beatific experience, we read and accord preëminence to one as head; surpassing all in either intellectual or physical superiority. Venus, the goddess of beauty and all its attributes, was esteemed by the ancients the most exquisite form of rounded symmetry, the perfect ideal of the harmony of shape. And Venus, too pure for earthly origin, sprang from the *sea* of crystsl charms and spotless white, a proper type of that which was to vanquish even Cupid in his strife for victory.

The siege of Troy forms the greater part of the books of Homer and the Æneid of Virgil. Yet the mighty stronghold, taking ten long years to be subdued, owed much of its surpassing strength to the god of the ocean. *Neptune*, with the assistance of Apollo, built a wall around the city and its neighboring precincts.

The all-inspiring fountain in the Ionian mount, whence poets drank, and muses quaffed, the source of mental power, the secret of their greatness, spouted forth from the burdened rock at the touch of proud Pegasus. His foot-print stamped immortality on those who might imbibe the gently flowing, spirit-like liquid.

How pleasing it is to peruse the ingenious narrative of the origin of frogs; when the fatal words of Leto: "May you live forever in that pool!" changed her tormentors into frogs, as a proper punishment for thus disturbing the pelucid water, and thereby preventing her from quenching the thirst of her distressed and suffering children.

> "Æternum stagno, discit, vivatis in isto;
> Eveniunt optata Deæ."

* The Lethean stream removed remembrances of sorrow, troubles, and afflicting cares, when once the unhappy victim of remorse plunged beneath its calm, unruffled waters.

What a sad, yet pleasing tale of sorrow was the melancholy fate of those unfortunates who were condemned to wander near the banks of the river Styx, till their allotted time expired.

It was not only the harsh sentence of the more feeble of mankind. The punishment, among the gods, for perjury, was to drink of this same river Styx, which instantly dulled their minds, and rendered them senseless for the weary period of one long year.

And when death claimed the body of a man, be it the remains of a mighty warrior, or the corpse of some forlorn, slain menial, how stern was father Charon for his obolus, as toll across the Styx and Acheron, ere rest could be obtained for those who had yielded up their spirits to the foe of life!

The Naiades, or water nymphs, presided over rivers, brooks and springs; while the Limniades, or lake nymphs, had the special charge of lakes and pools awarded to their safe keeping. The all-powerful and intrepid leader, Achilles, descended from Jupiter, and son of Thetis, herself goddess of the *sea*, was rendered invulnerable by being immersed, when a child, beneath the river Styx. His heel, the only portion of his body not thus covered by the shielding waters, did not escape in future years the skillful aim of his inveterate enemies.

We frequently read in mythology, of water being dashed

---

* "Lethe, the river of oblivion, rolls
Her watery labyrinth, whereof who drinks,
Forthwith his former state and being forgets,
Forgets both joy and grief, pleasure and pain."
*Paradise Lost*, b. vi.

upon some erring god, as a punishment, thereby instantly changing him by a transmigratory power to some inferior being. Actæon, following the promptings of curiosity, and urged on by the passionate feelings of an enamored lover, having surprised Artemis and her nymphs while bathing, was instantly transformed into a stag, *water* being sprinkled over him when captured. Midas, having experienced the misery of his gifted touch, wearied by the constant wretchedness of so unfortunate a power as he possessed, imploring Bacchus for some remedy that might at once free him from its future evils, was directed simply to wash in the river Pactolus.

What a touching story is that of Narcissus, who, when bereaved of his beloved sister, gazed for hours daily in the limpid stream, that he might but behold his own reflected image, which resembled her sweet face, and thus recall the happiest associations of purest friendship.

——— * Sed opaca fusus in herba
Spectat inexpleto mendacem lumine formam.
Perque occulos perit ipse suos.

The romantic account of Perseus and his mother cast upon the watery deep in a "crazy bark," rescued by the goddess, and conveyed to the Orgean shore, where he grew up to be one of the most interesting and captivating of fictitious characters, is a tale full of the marvelous, yet restrained within the bounds of possibility. Fascinating and instructive; imaginative, yet consistent.

The voyages of Jason and his faithful followers will be long remembered by the student of antiquity, and cherished by the lover of classic description and nautical adventures. It required the union of heaven and earth to produce *Oceanos* and the giants with fifty heads and one hundred hands. Pandora, the first woman, most fascinating in herself, yet possessing latent in her box all the evils that befall man in this life, was formed by Hephæstos, or Vulcan, out of earth and water. What poetry is there in the metamorphosis of the Heliades, transformed into poplars, their tears for their afflicted brother changing into amber as they fell into the river Eridanos.

When Psyche found herself deserted by the much loved Eros, alone, neglected and unhappy, she sought only for death amid the waters of the classic stream. Tantalus, so often quoted for his great, unceasing sufferings, was punished for

---

* Ovid, Met. 3.

his offenses by an insatiable thirst. Placed in the midst of the lake, he was permitted to see around him the beauteous, clear and limpid waters; his tormentors allowed him to feel the cool, refreshing touch of the lake. It rose up even to his chin, his lip; and when the mind was fixed on quenching parching thirst and thus alleviating untold agonies, at the last moment, straining every muscle to sip the nectar for a dying man, he failed, and was thus tantalized! It was only by turning a river into the stable of Augeas, that the mighty Hercules succeeded in cleansing the accumulation of the filth of 3,000 oxen for thirty years. There are many exquisite little stories of plaintive purport and beautiful sentiment pervading the narratives of classic deities, springing from streams presiding over fountains; punishing some by condemning them to remain near a gentle rivulet, thereby making use of water in every point of view.

Water, regarded in an historical light, affords much, indeed alike curious to the casual observer and instructive to the scholar in his researches. To the man of thought, a fact needs but to be mentioned, to call forth suggestive memories and philosophical deductions. Thus it is only necessary to mention that the turning point of Cæsar's great career; the conclusive period of his future life; whether he was to remain forever an obscure, unknown commander, in a native town, recognized as valorous only by the few who followed his own steps, was whether he should cross the Rubicon; dare the brave; meet the strong; vanquish conquerors; depose kings; mount to the loftiest eminence, and establish a new era for the historic page. After contending with hidden emotions, that for days had swayed him to and fro, he boldly plunged into the raging stream, and

"Primus in obliquum sonipes opponitur amnem,
Excepturus aquas: mollitum cetera rumpit
Turba vado faciles iam fracti flumina undas—
Cæsar ut adversam, superato gurgite ripam
Attigit, Hesperiæ vetitis et constitit arvis:
'Hic, ait. hic pacem temerataque jura relinquo;
Te, Fortuna, sequor'——."

When Archimedes was deputed to ascertain the specific gravity of the gold in a crown, to demonstrate its purity, he devised all methods for this purpose, but with ill success, and vain attempts. Nothing came to aid him in his philosophical investigations. At length, while bathing, we are informed, he noticed the gradual rise of the water on his entering the bath, and thus the happy " Eureka " was indebted to the accommodating element, which taking pity on the great philosopher, rose to elucidate the paradox, and relieve his mind of so perplexing a phenomenon, while he, in turn, for benefits received, formed the screw, that bears his name, likewise to elevate the subtle form, and refresh and give assistance to mankind in all his labors.

Napoleon's greatest feat in prowess, discipline and energy, has been considered to be his having crossed successfully the lofty Alps.

> * " The palaces of nature, whose vast walls
> Have pinnacled in clouds their snowy scalps,
> And throned eternity in icy halls
> Of cold sublimity, where forms and falls
> The avalanche—the thunderbolt of snow !"

The surmounting all the difficulties of the journey, and rendering submissive unto man the powerful opposing elements; the maintaining order in the midst of suffering, and when surrounded by a fearful chaos, all have joined to prove the man, the warrior, and the mighty leader of a warlike race of beings. But what was it that rendered what had been accomplished worthy to be classed among the wonders of the times ? What was it that secured renown and victory when many had displayed an equal zeal and overcome opposing obstacles, most harrassing and destructive in their deadly effects ; most woeful in their sad realities ? It was that Bonaparte subdued the snow clad peaks and ice-bound passes :

> Snow to the right of them;
> Snow to the left of them;
> Snow all in front of them !

While it rendered more facile the journeyings of the sleigh, and weary traveler, thereby a fit emblem of its peaceful efforts to assist, it stood its ground inch by inch against the onward movements of artillery and soldiers heavy armed, real harbingers of war, and all the necessary evils that ensue.

The lofty ascents and perilous marches of the regiments would have been as nothing, had not *snow* intervened with frigid, firm, and forcible impediments, and called forth all the discipline of an absolute sway to subdue the murmurs of the wasted army, and overcome the rebellion of subordinates, at their unexpected hardships.

---

* Childe Harold's Pilgrimage.—*Byron.*

Now falling stealthily in heavy flakes, to conceal from view the landmarks fixed, by which to make the tour; and as noiselessly, but with a subtle purpose, covering up the dead who had succumbed to feebler health and winter's powerful revenge. Now blinding each and every one by its heavy drapery, shrouding nature as an emblem for the approaching sacrifice of life, and piling up additional labor for the shovel and poor draught horse. Now, paling earth and enveloping the very clouds in white; a metaphysical expression of contrast with man's blackening deeds! The spotless heavens weeping for the loss of man, in pearly drops of chilling truth; all told but one sad tale of sorrow, woe, and future struggles. It was the vanquishing, or rather the permeating one of the Almighty's elements—water—that stamped Napoleon with renown, more than the controlling armies in the battle field, or overcoming nations in a foreign land.

The torrid zone, with all its withering heat, may be penetrated by the traveler in his paths of industry and deep research. The equator, with its scorching, red-hot line of solar concentration, may be crossed by seamen, and frequented by the pale-faced wanderer, on a visit to the very native, who both lives and experiences pleasure, while inhabiting the hottest region of the globe. All have easy access to the warmest latitudes of both the hemispheres; and nature seems to revel in luxuriant foliage and gaudy birds of many tints, where winter "never comes to rule the varied year,"* or cast one *cold* reflection on the happy holiday of flowers, fishes, fruits, flamingos, or the freedom of a fertile forest.

Agreeably to the wonderful, impartial law of compensation, we find insects that infest, but they may be destroyed. Reptiles may add dangers to the quiet calm of all around, but they may be avoided. The sun may pour down all his rays—parch the ground; shut up the heart of nature by his impetuous zeal; and drive all living creatures from his immediate presence. Still a shelter may be always found beneath the lofty pine, until forgiving night approaches with cool, refreshing shade, to elevate the soul and soothe the heated frame.

But when man, with his puny strength, seeks to penetrate the North and lay bare the hidden treasures of the polar region, what prevents his movements onward, and, with facile

---

* Thomson's Seasons.

stride, plants obstacles at every step? What, says Excelsior, can not be obtained by such means in the frigid zone? What, at his approach, lashes his bark with angry waves and mounts the prow of the little craft flowing with a maddened fury to the very stern, washing all before it to destruction? And now that man, still bent on moving forward, keeps his course, the mighty element assumes more hardened features, and, towering above his little craft, threatens instant ruin to all on board.

Icebergs, the lofty sentinels of the northern sea, moving to and fro with an irresistible power, keep off all those who are not "men of granite" from advancing farther toward the hidden mysteries of the frozen region! And when success attends man in his windings through the sinuosities of the chilly inlet, and his steps approach still nearer north, Nature comes again to aid in the resistance. The "hummochs" mount yet higher on the floes, exulting that a victim is to be encased, and all becomes as one; the masts and shrouds are coated with the sleet; the snow descends with that mysterious power, and naught remains to meet the view save the pale-faced, chilly monument of a former visitor—its own recorder.

Man may endure the scorching heat of the equator. The north pole, however, presents an opposition that is unequaled in its strength and durability. It is the agent, ice! Like man, the northern region places a barrier of cold reserve to all intruders, and shuts out those that would, from idle curiosity, penetrate beyond the calm exterior. But Nature, true in her characteristics to human nature—often her inferior—beneath that cold, impassive surface, possesses a warm and genial heart. Thus the Polar Sea, shut out from the mixed knowledge of a busy world, pulsates with warm emotions, sustaining many a weary inhabitant of the hidden waters; and, leaving the shores that encircle it with jealous seclusion, it sends forth toward the beaming light of day many a laughing ripple. As in the most northern ice-bound land we find refreshing springs of water, so in every character, however much degraded by a sinful life and fallen nature, we will find some latent spark, the remnant of a ray from the Sun of righteousness—some few pearly drops of priceless value from the Fountain of living waters!

It was the surmounting countless difficulties, and relying upon the merits of the compass, while entrusted to the ocean's wayward movements, that a Vasco de Gama discovered a path for commerce, which led to many followers ere long. It was the battling with the elements and living in the face of dangers, and braving the deep, when all but water had left Columbus to himself and a disaffected crew, that has named this favored country after so illustrious a man.

It was the great endurance of surpassing hardships when, amid the lonely glaciers,

> " O, Winter, ruler of the imperial year;
> Thy scatter'd hair with sleet-like ashes fill'd;
> Thy breath congeal'd upon thy lips; thy cheeks
> Fringed with a beard made white with other snows
> Than those of age; thy forehead wrapp'd in clouds,"*

that renders the name of Kane a synonym for energy and perseverance, the two great principles that urge man on to action of a noble character. All the powerful barriers of nature could not protect the English isle from Gallic tyranny; all the forces from the British kingdom could not keep off the approach of eager Frenchmen, rendered fiercer by the memory of Waterloo; but the gentle flowing of a mediating sea, intervening with a hidden depth of purpose, with persuasive eloquence, separates the usurper from his longed-for victim, and renders free a civilized community, destined to enlighten nations by its liberal form of government, and improve the morals of many a political body by the soundness of its ethics.

Venice, in former days annually wedded to the sea, to whom it owed so much for glory, riches, and surpassing rivalry, by her commerce took the lead, and for many a year was known to all surrounding powers under the amiable title of "Facile Princeps." Her graceful gondolas—those exquisite abodes of swan-like symmetry and easy gliding—and her islet residences leading down to the very waters of the Adriatic, struck man's imagination with a suggestive beauty that might well have inspired a Titian with sublimer colors, or called forth poetry from the Improvisatore. And even in the modern light of civilized humanity, her scenes recall so vividly the past, her palaces stand forth so exquisitely molded, as the fitting representatives of what has happened ages since, that we are told by one of thought and feeling that †"Venice is the only city that can yield the magical delights of solitude."

---

* Cowper's Task. Book 4th, line 120.

† B. D'Israelli.

"Oh! agony—that centuries should reap
No mellower harvest! Thirteen hundred years
Of wealth and glory turned to dust and tears;
And every monument the stranger meets
Church, palace, pillar, as a mourner greets."*

What period in the history of the colonial struggles in the American Revolution is more interesting to the student—more significant of the turning point of fate—more replete with facts of moment and proofs of firmness, fortitude and faith—than the great era in the life of Washington, when he crossed the Delaware amid the dangers of a wintry season, surrounded by difficulties and encompassed by prolonged privations? Speaking of this event in the following terms, Irving, in his classical biography, writes with eloquent enthusiasm, and states that "he himself crcssed with the rear-guard, on Sunday morning, and took up his quarters about a mile from the river, causing the boats to be destroyed and troops to be posted opposite the fords." He was conscious, however, as he said, "that, with his small force, he could make no great opposition should the enemy bring boats."†

Xerxes, as a great commander, receives the highest commendation of historians for his bright success, his happy power of subduing envy, and enforcing strict obedience. In the full list of his mighty exploits and most daring expeditions, we can find none recorded comparable with the vast, herculean feat of bridging over the Hellespont, that he might move on in regal magnificence as over a royal road, uninterrupted by an intervening water, thereby uniting both the Continents under his proud dominion. This wonderful work is the most durable record of the boldest of his schemes; the most successful of his adventurous undertakings. While his crossing the Hellespont, sacred to the Greeks, reflects credit, and indicates his prowess as a leader, his causing the sea to be lashed for contending against the power of his god-like attributes, points out the weakness of human nature in prosperity; the folly of an unconquered chief.

The story of Canute and his noble followers before the mighty inrolling of the waves that would not cease their movements even in the presence of a royal ruler, is replete with suggestive morals, and significant of the hollow boastings of the parasite. Though men, with souls, left to their own

* Byron's Ode to Venice.

† Irving's Life of Washingion. Vol. ii, p. 454.

choice, yielded up a ready and most cheerful homage to their king, and hastened to obey the slightest signal of command, the gentle ripple of *nature's* waters still kept on her course in spite of all the dictates of a man, though he were the head of all who breathed around him in an atmosphere of shallow worship.—*Medical and Surgical Reportor.*

